# Impact of Joint Commission International (JCI) Accreditation on Patient Satisfaction with Outpatient Departments: Comparative Cross-Sectional Study in Astana, Kazakhstan

**DOI:** 10.3390/ijerph23040473

**Published:** 2026-04-09

**Authors:** Kaisar Kudabayev, Aigul Ismailova, Kenesh Dzhusupov, Oxana Tsigengagel, Yerlan Naubetov, Bakhyt Yeleussizova, Yedil Omyrzakov

**Affiliations:** 1 Department of Epidemiology and Biostatistics, Astana Medical University, Astana City 010000, Kazakhstan; 2 Department of Public Health, International Higher School of Medicine, Bishkek City 750065, Kyrgyzstan; 3 Department of Radiology, “University Medical Center” Corporate Fund, Astana City 010000, Kazakhstan; 4 Department of Statistics, Turkestan City Polyclinic, Turkistan City 161200, Kazakhstan; 5 Department of Marketing, Suleyman Demirel University, Almaty City 050000, Kazakhstan

**Keywords:** hospital accreditation, Joint Commission International (JCI), customer satisfaction, outpatient care, healthcare quality improvement

## Abstract

**Highlights:**

**Public health relevance—How does this work relate to a public health issue?**

- This study evaluates whether international accreditation (JCI) is associated with better patient-reported satisfaction with outpatient services.
- Patient satisfaction reflects accessibility, communication, responsiveness, and perceived quality of care.

**Public health significance—Why is this work of significance to public health?**

- The study provides empirical evidence that JCI-accredited facilities demonstrate significantly higher patient satisfaction across all assessed domains.
- In the context of Kazakhstan’s ongoing health system reforms, these findings can inform national discussions on accreditation models and quality improvement strategies.

**Public health implications—What are the key implications or messages for practitioners, policymakers and/or researchers in public health?**

- Policymakers and healthcare administrators may consider international accreditation as a strategic investment for enhancing patient-centered care.
- Future public health research should explore the long-term outcomes, cost-effectiveness, and scalability of accreditation models.

**Abstract:**

The current study aimed to examine whether Joint Commission International (JCI) accreditation is associated with higher patient satisfaction. A cross-sectional, questionnaire-based comparative study was conducted between April and July 2025 in outpatient departments of one JCI-accredited hospital (University Medical Center) and two nationally accredited public polyclinics in Astana, Kazakhstan. The questionnaire was designed to assess satisfaction across four domains: communication, staff responsiveness, hospital environment, and perceived quality of care. The patients attending the JCI-accredited hospital demonstrated significantly higher satisfaction across all domains compared to those of nationally accredited hospitals (*p* < 0.01). The largest difference was observed in staff responsiveness. In the multivariable regression analysis, the accreditation status emerged as the strongest independent predictor of overall patient satisfaction score (β = 0.42; 95% CI: 0.31–0.53; *p* < 0.001), even after adjusting for age, gender, education, employment status, and prior hospital visits. Education level and previous hospital experience were modest yet statistically significant predictors, whereas age, gender, and employment status were not significant in the adjusted analyses. JCI accreditation was associated with higher patient satisfaction scores in outpatient care settings, indicating a positive relationship between accreditation status and patient-centered outcomes.

## 1. Introduction

Improving the quality and safety of healthcare services remains a global priority. A widely adopted strategy to promote continuous quality improvement is hospital accreditation. Healthcare accreditation programs are globally recognized for promoting patient safety and care quality by ensuring that procedures follow established standards [1,2,3]. Among international accreditation programs, the Joint Commission International (JCI) is one of the most recognized bodies, establishing standards focused on patient safety, clinical governance, risk management, and organizational leadership [1,4,5]. Hospitals that achieve JCI accreditation are expected to demonstrate compliance with rigorous, evidence-based standards and to engage in ongoing performance measurement and improvement [3,4].

Accreditation aims to enhance clinical outcomes, patient safety, and overall improvement in the quality of health services by establishing standardized protocols, promoting continuous quality improvement, strengthening staff training, and ensuring adherence to evidence-based practices. These mechanisms can translate into improved patient satisfaction by fostering more effective communication, reducing medical errors, enhancing responsiveness of care, and creating safer and more organized healthcare environments. Patient satisfaction assesses how well a service meets or exceeds patient expectations, impacting satisfaction, loyalty, and success [6,7,8]. It is measured by comparing service delivery to patient expectations; satisfaction becomes a central indicator of healthcare quality, reflecting patients’ perceptions of communication, responsiveness, environment, safety, and continuity of care [9]. As healthcare systems increasingly emphasize patient-centered care, satisfaction metrics are frequently incorporated into performance dashboards, public reporting systems, and reimbursement frameworks [2,5]. Therefore, evaluating whether accreditation translates into measurable improvements in patient satisfaction is important.

While accreditation standards emphasize structural and process improvements, such as standardized protocols, staff training, infection control, and quality monitoring, the extent to which these changes are perceived by patients remains inconclusive. Although some studies suggest that accredited hospitals demonstrate better organizational performance and safety culture, the evidence linking accreditation status directly to higher patient satisfaction is mixed [10]. Differences in institutional resources, case mix, staffing levels, and organizational culture may also influence patient perceptions independently of accreditation status [11].

Comparing patient satisfaction between JCI-accredited and nationally accredited hospitals provides an opportunity to assess whether international accreditation is associated with measurable differences in patient-reported experience. Such evidence can inform hospital administrators, policymakers, and accreditation bodies about the value of investing in accreditation programs and the areas in which accreditation may or may not influence patient-centered outcomes. Therefore, the aim of this study is to examine whether hospital accreditation is associated with differences in patients’ satisfaction levels. Specifically, this study seeks to determine whether patients treated in a JCI-accredited hospital report higher overall satisfaction compared with patients treated in non- accredited hospitals. In addition, the study aims to assess whether accreditation status is associated with differences in specific dimensions of patient satisfaction, such as communication, responsiveness of staff, hospital environment, and perceived quality of care, while accounting for the relevant demographic and clinical characteristics. We hypothesized that patients receiving care in JCI-accredited hospitals would report significantly higher overall satisfaction than patients receiving care in nationally accredited hospitals.

## 2. Methods

### 2.1. Design and Setting

This is a cross-sectional, comparative survey-based study designed to assess and compare patient satisfaction between JCI-accredited and nationally accredited hospitals in Astana. The study was conducted in the outpatient departments (OPDs) of selected public hospitals providing ambulatory care services, including University Medical Center, a JCI-accredited institute, and city polyclinics #4 and #9, nationally accredited institutes. The hospitals were selected based on their accreditation status, public sector affiliation, and provision of comparable outpatient services within the same urban setting. The selected facilities are among the largest providers of ambulatory care in Astana, serving diverse patient populations and offering a broad range of specialties. The data were collected from patients attending outpatient clinics for consultation, follow-up visits, or diagnostic services over a period of three months between April and July 2025. Patient satisfaction was measured immediately after the outpatient visit, with the participants completing the survey on-site following their consultation or service encounter to capture their real-time perceptions of care. The OPDs included various specialties, such as internal medicine, surgery, and other subspecialty clinics. Focusing on the outpatient setting enabled the evaluation of patient satisfaction related to ambulatory care services, including registration procedures, waiting time, communication with healthcare providers, and the overall experience within the OPD environment.

### 2.2. Ethical Approval

The study protocol was reviewed and approved by the Local Bioethical Committee at Astana Medical University in Astana (Approval No. 6, dated 24 September 2024). Prior to participation, all eligible individuals were provided with both written and verbal information outlining the purpose and procedures of the study. Written informed consent was obtained from each participant who agreed to take part in the survey. The participants were informed that their participation was entirely voluntary and that they could withdraw from the study at any time without any consequences to their care. No financial incentives or compensation were provided for participation. The study was conducted in accordance with the principles of the Declaration of Helsinki.

### 2.3. Study Population and Sample Size

The study population included adult patients attending the outpatient departments of selected JCI-accredited and nationally accredited hospitals in Astana during the study period. The sampling frame consisted of all adult patients visiting the outpatient departments of the selected hospitals during data collection hours. A consecutive sampling approach was used to recruit eligible participants, who were approached by trained research assistants who explained the study objectives and obtained written informed consent. Eligible patients were identified after completion of their outpatient visit and invited to participate using a uniform recruitment script. Before collection, training was provided for all research assistants to ensure consistency in recruitment procedures across sites. Differences in patient flow between sites were not formally controlled or adjusted for; however, data collection was conducted during comparable clinic operating hours and across similar outpatient services to minimize potential variability. The required sample size was calculated based on an expected prevalence (P) = 0.2, confidence level Z = 95%, and margin of error (d) = 0.05, resulting in a minimum sample size (N) of 246 participants. The participants completed the questionnaire anonymously, either digitally or on paper, and submitted their responses in sealed envelopes to maintain confidentiality.

**Inclusion Criteria:** Patients aged 18 years or older; attending the outpatient department for consultation, follow-up, or diagnostic services; and who completed their outpatient visit at the time of recruitment. Participants who were clinically stable and able to communicate in Kazakh or Russian and provided written informed consent were included in the study.

**Exclusion Criteria:** Patients younger than 18 years; those with documented cognitive impairment or inability to understand and respond to the questionnaire (e.g., disorientation, inability to follow simple instructions as assessed by the research assistant at the time of recruitment); those who were medically unstable, too ill to participate, or declined to provide informed consent; and patients who had already participated in the study during the data collection period were excluded.

### 2.4. Variables

The primary independent variable was hospital accreditation status (JCI accredited vs. non-accredited). The primary dependent variable was the overall patient satisfaction score. The secondary outcomes included the satisfaction scores across specific domains (communication, responsiveness, environment, and perceived quality of care). The covariates included demographic and clinical characteristics.

### 2.5. Data Collection Tool

The data were collected using a structured, self-administered questionnaire mostly adapted from Harazni et al., 2025, which is validated patient satisfaction instrument [12]. The questionnaire consisted of two sections:

*Sociodemographic and clinical characteristics*, including age, gender, education level, marital status, and previous hospitalization history.

*Patient satisfaction domains*, covering communication with healthcare providers, responsiveness of staff, hospital environment, perceived quality of care, discharge information, and overall satisfaction. The patient satisfaction section comprised a total of 24 items distributed across domains as follows: communication with healthcare providers (5 items; e.g., “The healthcare provider clearly explained my condition and treatment”), responsiveness of staff (4 items; e.g., “Staff responded promptly to my requests”), hospital environment (4 items; e.g., “The hospital environment was clean and comfortable”), perceived quality of care (4 items; e.g., “I received high-quality medical care”), discharge information (3 items; e.g., “I was given clear instructions upon discharge”), and overall satisfaction (4 items; e.g., “Overall, I am satisfied with the care I received”).

The responses were measured using a five-point Likert scale ranging from 1 (strongly disagree/very dissatisfied) to 5 (strongly agree/very satisfied). The domain scores were calculated by taking the mean of the items within each domain, with higher scores indicating greater satisfaction. The overall satisfaction score was computed as the mean of all items across domains. Missing responses were handled by calculating domain scores if at least 75% of items within the domain were completed. The questionnaire was available in Kazakh and Russian. A forward–backward translation process was performed by two independent qualified bilingual translators, with discrepancies resolved through consensus, to ensure linguistic validity. For the purpose of statistical analysis, the Likert-scale items were treated as continuous variables when computing the mean domain and overall scores, while the individual item responses retained their ordinal nature.

### 2.6. Validation and Reproducibility

To ensure the validity and reliability of the survey, a pilot study was conducted with 20 participants from of the selected outpatient departments prior to the main data collection. The aim of the pilot study was to test the clarity and relevance of the questionnaire items, as well as to assess the feasibility of the data collection process. During the pilot phase, participants were asked to complete the same survey that would be used in the main study. Their feedback on the survey’s wording, structure, and ease of completion was collected to refine the instrument and address any ambiguities. Based on the results of the pilot study, minor linguistic adjustments were made to the questionnaire to improve clarity and ensure that the items accurately captured the dimensions of patient satisfaction. In addition, Cronbach’s alpha coefficient was calculated to test the internal consistency of the questionnaire. A value of 0.80 was obtained, indicating a high level of reliability and internal consistency, which is considered acceptable for the measurement of patient satisfaction in healthcare settings.

### 2.7. Data Storage

To ensure the confidentiality, integrity, and security of the collected data, all study-related data were stored in compliance with the relevant ethical guidelines and regulations governing patient privacy and data protection. The collected data were anonymized to prevent identification of participants. Each participant was assigned a unique identifier code, which was used on all study documents and digital records to ensure confidentiality. Hard copies of the completed questionnaires were securely stored in locked filing cabinets within a restricted access area at the research office. The digital data, including the electronic copies of the questionnaires and database entries, were stored on password-protected computers. Access to both the physical and digital data was limited to the research team members involved in data analysis and reporting.

### 2.8. Data Analysis

The data were entered and analyzed using Microsoft Excel and statistical software (SPSS version 25.0). Descriptive statistics were used to summarize the participant characteristics and satisfaction scores.

The continuous variables were presented as means and standard deviations, while the categorical variables were summarized using frequencies and percentages. Comparisons between the accredited and non-accredited hospitals were performed using independent sample *t*-tests for the continuous variables and chi-square tests for the categorical variables. Independent samples *t*-tests were also used to assess the differences in overall patient satisfaction across demographic subgroups. Given the number of subgroup comparisons performed, the results should be interpreted with caution, as no formal adjustment for multiple testing was applied. A multiple linear regression analysis was conducted to determine whether accreditation status was an independent predictor of patient satisfaction after adjusting for potential confounders. A *p*-value of less than 0.05 was considered statistically significant.

## 3. Results

### 3.1. Reliability Analysis

The internal consistency of the survey instrument was assessed using Cronbach’s alpha coefficients based on the full study sample (*n* = 311). The overall scale demonstrated good reliability (α = 0.80). The domain-specific analysis showed acceptable-to-good internal consistency across all subscales: communication (α = 0.81), responsiveness (α = 0.83), hospital environment (α = 0.78), and perceived quality of care (α = 0.80). All values exceeded the commonly accepted threshold of 0.70, supporting the reliability of the instrument.

### 3.2. Demographic Data

Approximately 400 people were invited to participate, of whom 311 participated in the study, from both the JCI-accredited and nationally accredited outpatient departments, resulting in a response rate of 78%. The participants’ ages ranged from 18 to 75 years, with a mean age of 42.6 ± 14.3 years. The majority of participants (58%) were aged between 30 and 50 years, and 178 participants (57.2%) were female and 133 participants (42.8%) were male. A total of 112 participants (36%) had completed secondary education, 148 participants (47.6%) held a university degree, and 51 participants (16.4%) had postgraduate or professional qualifications. Most participants were employed (54.7%), followed by students (12.2%), unemployed individuals (18%), and retirees (15.1%). The marital status data showed that 62.4% were married, 28.3% were single, and 9.3% were divorced or widowed. About 71% of participants reported previous experience with hospital outpatient services, whereas 29% were visiting the hospital for the first time [Table 1]. These findings reflect a diverse outpatient population in terms of age, gender, education, employment, and prior healthcare exposure, providing a broad and heterogeneous sample for evaluating patient satisfaction across JCI-accredited and nationally accredited hospitals. The demographic characteristics were stratified by hospital type (JCI accredited vs. nationally accredited) to assess the baseline differences between groups, and no substantial imbalances were observed across the key variables. In addition, the employment and education distributions were comparable across sites, and no statistically significant differences were observed between groups.

### 3.3. Patient Satisfaction Results

Patient satisfaction was assessed across four key domains: communication with healthcare providers, responsiveness of staff, hospital environment, and perceived quality of care. Table 2 summarizes the mean scores for each domain at the JCI-accredited and nationally accredited outpatient departments. However, the patients attending the JCI-accredited hospital reported consistently higher satisfaction across all domains. The highest satisfaction was observed for communication with healthcare providers (mean 4.35 ± 0.48), followed closely by perceived quality of care (mean 4.30 ± 0.50). The responsiveness of staff showed the largest difference between the JCI-accredited (4.28 ± 0.53) and nationally accredited hospitals (3.65 ± 0.67), suggesting the impact of accreditation on timely and attentive patient service. The hospital environment also scored higher at the JCI-accredited hospital (4.21 ± 0.55 vs. 3.76 ± 0.61, *p* = 0.002), indicating improved facilities and cleanliness. Also, discharge information was included as part of the quality of care domain, and showed a high satisfaction score at the JCI-accredited hospital; consequently, an overall much higher satisfaction score across all items was demonstrated for the JCI-accredited hospital compared to its nationally accredited counterparts.

### 3.4. Demographic Data and Patient Satisfaction

The influence of demographic characteristics on overall patient satisfaction was evaluated using both a descriptive analysis and inferential statistics. Table 3 presents the mean overall satisfaction scores across the key demographic variables. For age, patients aged 30–50 years reported slightly higher satisfaction (mean 4.10 ± 0.55) compared to younger (18–29 years, mean 3.95 ± 0.60) and older patients (51–75 years, mean 4.05 ± 0.58), though the differences were not statistically significant (*p* = 0.12). Female participants reported marginally higher satisfaction (mean 4.05 ± 0.57) than male participants (mean 3.98 ± 0.60), but this difference was also not statistically significant (*p* = 0.18).

For education level, participants with secondary education reported a mean satisfaction of 3.92 ± 0.61, those with university education 4.08 ± 0.56, and participants with postgraduate or professional education 4.12 ± 0.53, with the overall differences being statistically significant (*p* = 0.03). Regarding employment status, employed participants reported a mean satisfaction of 4.07 ± 0.56, students 4.05 ± 0.58, unemployed 3.93 ± 0.61, and retired participants 3.95 ± 0.59; these differences were not statistically significant (*p* = 0.09). Finally, participants with prior hospital visits reported significantly higher satisfaction (mean 4.08 ± 0.56) than first-time visitors (mean 3.88 ± 0.60, *p* = 0.01).

### 3.5. Analysis of Predictors of Patient Satisfaction

In order to determine whether hospital accreditation independently predicts overall patient satisfaction, a multiple linear regression analysis was conducted, adjusting for key demographic variables including age, gender, education level, employment status, and previous hospital visits. The results, shown in Table 4, indicate that hospital accreditation status was a significant independent predictor of patient satisfaction. For instance, patients attending the JCI-accredited hospital reported higher satisfaction scores compared to those at nationally accredited hospitals (β = 0.42; 95% CI: 0.31–0.53; *p* < 0.001), even after controlling for demographic factors. Among the demographic variables, education level (β = 0.15; 95% CI: 0.02–0.28; *p* = 0.02) and previous hospital visits (β = 0.18; 95% CI: 0.05–0.31; *p* = 0.01) were also significant predictors of higher patient satisfaction. Age, gender, and employment status did not show significant associations in the adjusted model (*p* > 0.05). The regression model accounted for 32% of the variance in overall patient satisfaction (R^2^ = 0.32; adjusted R^2^ = 0.30; F (6, 304) = 23.5; *p* < 0.001). A diagnostic assessment indicated no major violations of regression assumptions, with the residuals approximately normally distributed and homoscedastic; there was also no concern of multicollinearity, as all values were low.

### 3.6. Covariate Analysis

The effect of hospital accreditation on patient satisfaction across different patient subgroups was analyzed by age, gender, education level, and previous hospital experience. Covariate-adjusted comparisons were conducted using an ANCOVA, with hospital accreditation as the fixed factor and age, gender, education, employment status, and prior hospital visits as the covariates. The *p*-values represent the adjusted difference in patient satisfaction between the JCI-accredited and nationally accredited hospitals within each subgroup. For age, patients were divided into three age groups: 18–29, 30–50, and 51–75 years. After adjustment for the covariates, the satisfaction scores remained significantly higher for the JCI-accredited hospital across all age groups (*p* < 0.01). The largest difference was observed in the 30–50 age group, with an adjusted mean satisfaction score of 4.33 for the JCI-accredited hospital versus 3.76 for the nationally accredited hospitals. As for gender, both male and female patients reported higher satisfaction with the JCI-accredited hospital; however, after adjusting for the covariates, the difference between genders was not statistically significant (*p* = 0.42). This indicates that gender did not influence overall satisfaction. In addition, patients with secondary, university, and postgraduate education all reported slightly higher satisfaction with the JCI-accredited hospital. However, the covariate-adjusted analysis showed that education level was not a significant predictor of satisfaction (*p* = 0.08), suggesting that the positive effect of accreditation is independent of patients’ educational background. Lastly, patients with prior hospital visits and first-time visitors both showed significantly higher satisfaction with the JCI-accredited hospital (*p* < 0.01). The covariate adjustment confirmed that previous hospital experience remained a modest, but significant predictor (*p* = 0.01) [Table 5]. Assumption checks indicated that the residuals were approximately normally distributed, the variances were homogeneous, and there were no significant interactions between the covariates and hospital type, supporting the validity of the ANCOVA comparisons. Also, the subgroup and covariate-adjusted analyses demonstrated that hospital accreditation has a consistent and significant positive effect on patient satisfaction across age and prior hospital experience. Gender and education level were not significant predictors, suggesting that the benefits of accreditation are independent of these demographic factors.

## 4. Discussion

The current study aimed to compare patient satisfaction between JCI-accredited and nationally accredited outpatient departments in Astana, Kazakhstan, with a focus on understanding the role of hospital accreditation in influencing patient perceptions of care quality. The findings reveal several key insights: the JCI-accredited hospital consistently demonstrated higher patient satisfaction scores across all domains, including communication, responsiveness, hospital environment, and perceived quality of care. However, given the cross-sectional design of this study, these findings should be interpreted as associations rather than evidence of causality. Additionally, hospital accreditation was found to be the strongest independent predictor of overall patient satisfaction, even after adjusting for demographic variables, such as age, gender, education level, and previous hospital visits, within the limitations inherent to a cross-sectional analytical framework.

The current findings align with previous studies that have shown hospital accreditation is associated with improved patient outcomes and satisfaction. For instance, a systematic review by Alkhenizan and colleagues et al. found that accreditation programs, such as those led by JCI, are associated with higher quality of care, as measured by patient satisfaction, safety, and clinical outcomes [13]. The results corroborate these findings, with patients at the JCI-accredited hospital reporting significantly higher satisfaction scores across multiple domains. Specifically, the responsiveness of staff showed the largest difference between the JCI-accredited and nationally accredited hospitals, which is consistent with the findings of Sack et al. (2011), who reported that accreditation programs are associated with improvements in service delivery and staff performance [14]. In addition, a cross-sectional study in a UAE tertiary hospital reported that repeated JCI surveys were associated with improved medication safety practices, strengthened safety culture, and enhanced healthcare processes, supporting the broader system-level implications of JCI accreditation [15].

Moreover, our study found that hospital accreditation was a significant predictor of satisfaction, even when adjusting for demographic characteristics, such as age, gender, and education level. Nevertheless, this observed relationship should not be interpreted as causal, as unmeasured confounding variables may influence both accreditation status and patient satisfaction outcomes. On the contrary, a study by Heuer et al., that investigated the relationship between hospital accreditation and patient satisfaction reported no relationship between the two [16]. This discrepancy could be influenced by regional differences, the accreditation status of the hospitals, and the specific service attributes assessed. Additionally, country-specific factors may play a role. For example, the current study investigated hospitals in Kazakhstan, which operate within a different healthcare environment compared to those in the USA, which was the setting studied by Heuer et al. However, a systematic review by Greenfield and colleagues that investigated the impact of accreditation on service quality concluded that accreditation programs can enhance the quality of care provided, which may be associated with patients’ satisfaction [17]. Therefore, the emphasis on structured procedures and continuous quality improvement in accredited hospitals might contribute to a better overall patient experience, although this interpretation remains inferential due to the study design.

Unlike several previously published studies that suggested that patient demographics, such as age, gender, and education level, may influence satisfaction [9,18,19], the analysis in the present study shows that after adjusting for other variables, gender and education level did not significantly impact overall satisfaction. This is in line with Jha et al., who studied patients’ perceptions of hospital care in the US and reported that demographic variables had limited predictive value for patient satisfaction when hospital-level factors were accounted for [20].

In contrast, age and previous hospital visits were found to have a modest impact on satisfaction. Older patients and those with previous hospital experience reported slightly higher satisfaction, which may reflect their greater familiarity with healthcare services and a higher baseline expectation for care quality. Hence, it is important to note that previous hospital experience was a significant predictor of satisfaction, with patients who had visited hospitals before reporting higher satisfaction with both accredited and non-accredited settings. This finding is in agreement with the findings reported by Bleich et al., who studied the relationship between satisfaction with the healthcare system and patient experience, and suggested that prior healthcare exposure leads to higher patient expectations, which in turn could influence satisfaction levels [21].

However, we believe that the current findings have important implications for healthcare administrators and policymakers. Given that hospital accreditation is associated with higher patient satisfaction scores, healthcare institutions might consider investing in accreditation programs as a strategy to improve care quality and patient perceptions. Furthermore, accreditation bodies like the JCI could consider emphasizing areas such as staff responsiveness and environmental factors, which were the most strongly associated with patient satisfaction in this study. The results also suggest that hospitals with higher levels of accreditation may be associated with better patient satisfaction, and also potential improvements in operational efficiency and clinical outcomes. At a systems level, accreditation processes may also reinforce institutional practices, such as patient safety protocols, including medication safety systems, continuous quality monitoring, and organizational culture improvements. However, these broader system-level implications should be interpreted cautiously and in conjunction with other structural and organizational determinants of healthcare quality.

## 5. Limitations

Although this study provides valuable insights into the relationship between hospital accreditation and patient satisfaction, there are several drawbacks. First, the cross-sectional nature of the study means that causality cannot be established; therefore, the observed relationships should be interpreted as associative rather than causal. Longitudinal studies could help clarify whether improvements in accreditation lead to sustained increases in patient satisfaction over time.

Additionally, while the study controlled for demographic factors, other potential confounders, such as patient health status or clinical outcomes, were not assessed. Important unmeasured variables, including disease severity, waiting times, staffing levels and ratios, and provider workload, may have influenced patient satisfaction and could partially explain the observed differences between hospital types. Thus, residual confounding may be present, and research into how clinical factors interact with accreditation status to influence patient satisfaction is warranted.

Furthermore, the use of non-random sampling of both patients and hospitals may have introduced selection bias, potentially limiting the representativeness of the study population and affecting the generalizability of the findings. In addition, facility-level confounding factors, such as hospital size, resource availability, organizational structure, leadership practices, and institutional quality improvement capacity, were not explicitly adjusted for, and may have influenced both the accreditation status and patient satisfaction outcomes.

Another limitation is that this study was conducted only in Astana, Kazakhstan, which may limit the generalizability of the results to other regions or countries. Cultural and healthcare system differences could influence how accreditation is perceived by patients and its impact on satisfaction. Future research should consider multi-center and international comparative studies to better understand the consistency of these associations across diverse healthcare systems and settings.

## 6. Conclusions

This study demonstrates an association between hospital accreditation and higher patient satisfaction in outpatient settings. The JCI-accredited hospital consistently outperformed the nationally accredited hospitals across key satisfaction domains. However, these findings should be interpreted with caution, as the cross-sectional design precludes causal inference and the observed associations may be influenced by unmeasured and facility-level confounding factors.

While demographic factors like age and prior hospital experience showed some influence on satisfaction, hospital accreditation status was the strongest associated factor. From a clinical and practical perspective, these findings highlight the potential value of accreditation frameworks for supporting improvements in patient-centered care, service delivery processes, and perceived quality of care. However, accreditation alone may not be sufficient to drive optimal patient outcomes.

These findings suggest that accreditation programs may be considered as one of several strategies to support improvements in patient satisfaction; however, the results should be interpreted with caution given the cross-sectional design and the potential for residual confounding. Healthcare policymakers and administrators should consider accreditation alongside other system-level interventions, such as workforce strengthening, reduction in waiting times, enhancement of patient safety practices, and optimization of care delivery processes, to achieve meaningful and sustained improvements in patient satisfaction.

## Figures and Tables

**Table 1:** Demographic data of study participants.

Characteristic	Frequency (*n*)	Percentage (%)
**Age (years)**		
18–29	74	23.8
30–50	180	57.9
51–75	57	18.3
**Gender**		
Female	178	57.2
Male	133	42.8
**Education Level**		
Secondary	112	36.0
University	148	47.6
Postgraduate/Professional	51	16.4
**Employment Status**		
Employed	170	54.7
Student	38	12.2
Unemployed	56	18.0
Retired	47	15.1
**Marital Status**		
Married	194	62.4
Single	88	28.3
Divorced/Widowed	29	9.3
**Previous Hospital Visits**		
Yes	221	71.1
No	90	28.9

**Table 2:** Patient satisfaction scores for JCI-accredited vs. nationally accredited hospitals (*n* = 311).

Satisfaction Domain	JCI Accredited (*n* = 130, Mean ± SD)	Nationally Accredited (*n* = 181, Mean ± SD)	*p*-Value
Communication with Providers	4.35 ± 0.48	3.82 ± 0.60	<0.001
Responsiveness of Staff	4.28 ± 0.53	3.65 ± 0.67	<0.001
Hospital Environment	4.21 ± 0.55	3.76 ± 0.61	0.002
Perceived Quality of Care	4.30 ± 0.50	3.79 ± 0.63	<0.001

**Table 3:** Overall patient satisfaction by demographic characteristics.

Subgroup	JCI Accredited (Adjusted Mean ± SE)	Nationally Accredited (Adjusted Mean ± SE)	*p*-Value *
**Age**			
18–29	4.05 ± 0.06	3.87 ± 0.07	0.008
30–50	4.33 ± 0.05	3.76 ± 0.06	<0.001
51–75	4.18 ± 0.07	3.81 ± 0.08	0.002
**Gender**			0.42
Female	4.31 ± 0.05	3.78 ± 0.06	-
Male	4.28 ± 0.06	3.77 ± 0.07	-
**Education Level**			0.03
Secondary	4.10 ± 0.07	3.79 ± 0.08	-
University	4.35 ± 0.05	3.80 ± 0.06	-
Postgraduate/Professional	4.38 ± 0.06	3.85 ± 0.07	-
**Employment Status**			0.09
Employed	4.32 ± 0.05	3.79 ± 0.06	-
Student	4.28 ± 0.07	3.75 ± 0.08	-
Unemployed	4.15 ± 0.06	3.72 ± 0.07	-
Retired	4.18 ± 0.07	3.74 ± 0.08	-
**Previous Hospital Visits**			0.01
Yes	4.34 ± 0.05	3.82 ± 0.06	-
No	4.2 ± 0.07	3.70 ± 0.08	-

* *p*-Value less than 0.05 was considered as statistically significant.

**Table 4:** Multiple linear regression analysis of predictors of overall patient satisfaction.

Predictor Variable	B	SE B	β Coefficient	95% CI	*p*-Value	VIF
Constant	1.25	0.32	-	0.62–1.88	<0.001	-
Hospital Accreditation	0.42	0.056	0.42	0.31–0.53	<0.001	1.25
Age (Years)	0.03	0.041	0.03	−0.05–0.11	0.45	1.10
Gender (Female vs. Male)	0.04	0.052	0.04	−0.06–0.14	0.42	1.01
Education Level	0.15	0.066	0.15	0.02–0.28	0.02	1.30
Employment Status	0.06	0.056	0.06	−0.05–0.17	0.27	1.22
Previous Hospital Visits	0.18	0.042	0.18	0.05–0.31	0.01	1.18

**Table 5:** Adjusted mean satisfaction scores by subgroup (ANCOVA).

Subgroup	JCI Accredited (Adjusted Mean ± SE)	Nationally Accredited (Adjusted Mean ± SE)	*p*-Value
**Age**			
18–29	4.05 ± 0.06	3.87 ± 0.07	0.008
30–50	4.33 ± 0.05	3.76 ± 0.06	<0.001
51–75	4.18 ± 0.07	3.81 ± 0.08	0.002
**Gender**			0.42
Female	4.31 ± 0.05	3.78 ± 0.06	
Male	4.28 ± 0.06	3.77 ± 0.07	
**Education Level**			0.08
Secondary	4.10 ± 0.07	3.79 ± 0.08	
University	4.35 ± 0.05	3.80 ± 0.06	
Postgraduate/Professional	4.38 ± 0.06	3.85 ± 0.07	
**Previous Hospital Visits**			0.01
Yes	4.34 ± 0.05	3.82 ± 0.06	
No	4.21 ± 0.07	3.70 ± 0.08	

## Data Availability

Data will be made available upon request from the corresponding author due to privacy reasons.
